# Research advance on cold tolerance in chrysanthemum

**DOI:** 10.3389/fpls.2023.1259229

**Published:** 2023-09-27

**Authors:** Qingbing Chen, Kang Gao, YuRan Xu, YaHui Sun, Bo Pan, Dongliang Chen, Chang Luo, Xi Cheng, Hua Liu, Conglin Huang

**Affiliations:** ^1^ Beijing Engineering Research Center of Functional Floriculture, Institute of Grassland, Flowers and Ecology, Beijing Academy of Agriculture and Forestry Sciences, Beijing, China; ^2^ College of Architecture, North China University of Water Resources and Electric Power, Zhengzhou, China

**Keywords:** phenotype, physiological mechanism, the forward genetics, molecular mechanism, breeding

## Abstract

Chrysanthemums are one of the top ten most well-known traditional famous flowers in China and one of the top four cut flowers worldwide, holding a significant position in landscape gardening. The cold temperatures of winter restrict the cultivation, introduction, and application of chrysanthemum, resulting in high costs for year-round production. This severely impacts the ornamental and economic value of chrysanthemum. Therefore, research on cold tolerance is of vital importance for guiding chrysanthemum production and application. With the development of genomics, transcriptomics, metabolomics, and other omics approaches, along with high-throughput molecular marker technologies, research on chrysanthemum cold tolerance has been continuously advancing. This article provides a comprehensive overview of the progress in cold tolerance research from various aspects, including chrysanthemum phenotype, physiological mechanisms, the forward genetics, molecular mechanisms, and breeding. The aim is to offer insights into the mechanisms of cold tolerance in chrysanthemum and provide reference for in-depth research and the development of new cold tolerance chrysanthemum varieties.

## 1 Introduction

Chrysanthemum (*Chrysanthemum* × *morifolium* Ramat.), a perennial herb of the *chrysanthemum* genus in the Asteraceae family, has a history of 4,000 years in Chinese written records (Zhang and Dai, 2013) and a cultivation history of more than 3,000 years (Zhang, 2017), with more than 3,000 varieties (Wang, 2020). Chrysanthemum is one of the top ten most well-known flowers in China. With a graceful posture, bright colours and pleasant flowers, it has high ornamental value. In addition, it also has edible, tea and medicinal functions. In ornamental plants, low-temperature stress not only affects their ornamental traits, such as flower shape and colour but also reduces their ornamental quality (Li et al., 2008), and annual production is affected by the restriction of their growth and development (Ao et al., 2019). In addition, the winter production of chrysanthemums in most areas requires the use of facilities for heating cultivation, which greatly increases production costs, especially in the environment of northern facilities. There is a low-temperature “rosette” phenomenon in chrysanthemum production (Li et al., 2015), which seriously affects their ornamental value and economic benefits (Li et al., 2010). To date, studies on cold tolerance have been reported for a variety of plants, and plant cold tolerance is considered a complex quantitative trait (Guy, 2003; Wang et al., 2016; Zhang et al., 2018). Research on the cold tolerance in chrysanthemum has focused on evaluation systems (Anderson and Gesick, 2004; Xu and Chen, 2008); physiological, biochemical (Li et al., 2013; Wang et al., 2014), and genetic mechanisms (Ma, 2017; Chi et al., 2018); key gene mining (Chen et al., 2012; Ren et al., 2014); germplasm innovation (Zhang X. J. et al., 2011; Chen et al., 2012; Chi et al., 2018) and other relevant information.

**Figure 1:**
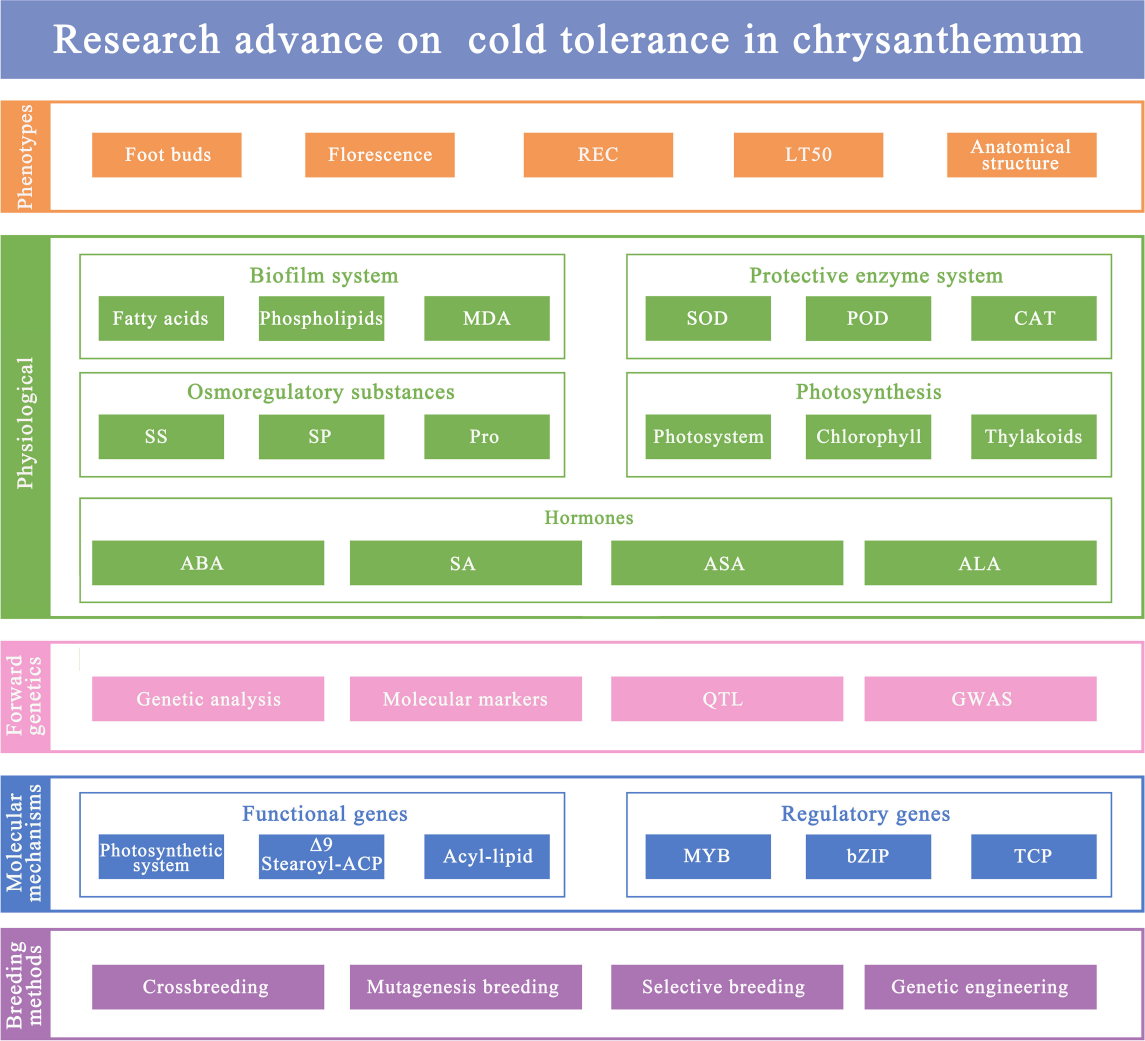
Review framework of cold cold tolerance in chrysanthemum.

This article provides a comprehensive review of the main cold tolerance evaluation indicators for chrysanthemum from both the phenotypic and physiological aspects. It also summarizes the existing research progress on the genetic and molecular mechanisms in chrysanthemum cold tolerance. Additionally, the breeding methods for cold resistant chrysanthemum are outlined (**Figure 1**). The aim is to offer a scientific basis for promoting excellent chrysanthemum varieties in cold regions, and to lay the foundation for further research into the mechanisms of chrysanthemum cold tolerance and the development of new cold resistant varieties.

## 2 Research advance on the phenotypes of cold tolerance in chrysanthemum

Low temperature is one of the main environmental factors affecting plant growth and development (Petrov et al., 2016; Xin et al., 2015), and low-temperature stress can result in phenotypic changes in plants. Many studies have analysed and evaluated their cold tolerance in chrysanthemum from the perspective of the foot bud (Anderson and Gesick, 2004), florescence (Li et al., 2013), relative conductivity (Li et al., 2009), semilethal temperature (Shi, 2013) and morphological structure (Xu et al., 2009).

### 2.1 Foot buds

Chrysanthemums are perennial herb flowers. After flowering, the ground part of the chrysanthemum withers, and foot buds sprout from the base to overwinter. The cold tolerance of the foot buds not only determines whether the chrysanthemum can safely overwinter but also affects the reproduction coefficient of the plant in the next year. Many studies have evaluated cold tolerance chrysanthemum mainly based on the number of foot buds and their growth. Anderson and Gesick (2004) found that the more foot buds that perennial overwintering roots had, the stronger the low-temperature tolerance of the variety was. Anderson and Gesick (2004) also judged the cold tolerance of different varieties by counting the number of foot buds in the ‘Tianjian’ autumn chrysanthemum. Zhang and Zhang (2014) used 15 groundcover chrysanthemums from the ‘Jinling’, ‘Zhongshan’ and ‘Yuhua’ series as experimental materials and evaluated their cold tolerance in terms of the frost damage grade of the petals and leaves of each variety, combined with the number of germinated foot buds in early spring. The petals of the ‘Yuhua’ series had the strongest cold tolerance, and the number of foot buds that germinated in early spring was also the highest. In addition, Kim and Anderson (2006) determined the cold tolerance of isolated foot buds and whole plants by two low-temperature treatment methods and established a method to evaluate cold tolerance in chrysanthemum. However, Li et al. (2014) found that the root rate, average number of roots and average root length of the foot buds of the groundcover chrysanthemum ‘Wanfen’ were the best when the cold storage temperature was 4°C and the cold storage time did not exceed 4 weeks, which allowed indirect evaluation of the cold tolerance of ‘Wanfen’. In conclusion, the foot bud is an important phenotypic trait in the study of cold tolerance in chrysanthemum.

### 2.2 Florescence

The florescence is one of the most important phenotypic traits of chrysanthemum and can be divided according to natural florescence into spring chrysanthemum (late April to late May), summer chrysanthemum (late May to August), autumn chrysanthemum (late October to late November), and cold chrysanthemum (early December to February of the following year) (Wan, 2013). Related studies have found a correlation between chrysanthemum florescence and cold tolerance. In particular, Li et al. (2013) used early autumn chrysanthemums ‘Taiping’s Snare Drum’ and ‘Jin Fengling’ and late autumn chrysanthemums ‘Starlight Bright’ and ‘Mobao’ as materials and found that the unsaturated fatty acid contents of ‘Taiping’s Snare Drum’ and ‘Starlight Bright’ were significantly higher than those of the other two varieties at 16°C and 5°C. At -4°C and -8°C, the unsaturated fatty acid content of the early flowering variety ‘Jinfengling’ was significantly lower than that of the other three varieties, and the saturated fatty acid content was the opposite. It was speculated that the flowering period of autumn chrysanthemums had a certain relationship with the unsaturation of fatty acids and cold tolerance. Wang et al. (2014) performed cluster analysis of nine autumn chrysanthemum varieties and found that early flowering varieties had stronger cold tolerance, while late flowering varieties had weaker cold tolerance, and the flowering date was related to the cold tolerance of the chrysanthemums to some extent. Li (2010) analysed the cold tolerance of two different cut chrysanthemum varieties, ‘Cold Purple’ and ‘Cold Yellow’, and found that when the temperature was reduced to 9°C or 6°C, physiological obstacles began to appear in the flowering period. However, Xu and Chen (2008) ranked the cold tolerance of eight different chrysanthemums by comparing the semilethal temperatures (LT50) and found that there was no significant correlation between cold tolerance and florescence. In summary, there is a certain relationship between the florescence and cold tolerance in chrysanthemum, but the variety has a certain impact on this relationship, and the correlation between florescence and cold tolerance needs to be further studied.

### 2.3 Relative conductivity and semilethal temperature

When plants are under low-temperature stress, it is mainly manifested as reduced fluidity, enhanced permeability, and different degrees of changes in the structure and composition of the cell plasma membrane, resulting in different degrees of electrolyte exosmosis (Xu, 2012). Therefore, relative conductivity (REC) can be used as an evaluation indexes of plant cold tolerance. Recently, the REC index has been directly used to evaluate the cold tolerance of many plants (Zeng et al., 2016; Wei et al., 2017; Yang et al., 2017), but it has rarely been used to study cold tolerance in chrysanthemum. Li et al. (2009) subjected autumn white chrysanthemum to low-temperature exercise at 4°C and low-temperature stress at -5°C and found that the REC of chrysanthemum leaves after low-temperature exercise was significantly lower than that of the control, indicating that low-temperature exercise can enhance the cold tolerance of chrysanthemum and alleviate the degree of damage to the chrysanthemum leaf film.

In the study of chrysanthemum cold tolerance, the LT50, calculated by combining the REC with a logistic equation, is generally used as an important index to evaluate cold tolerance (Liu et al., 1985; Wang, 1987). Shi (2013) calculated LT50 by measuring the membrane lipid physiological indices of leaves and roots under low-temperature stress, and a comprehensive evaluation of the cold tolerance of different autumn chrysanthemum varieties was conducted. Xu and Chen (2008) measured the REC of leaves of different chrysanthemum varieties during the whole process of the temperature drop to rise in autumn and winter, recorded and calculated changes in LT50 values, and found that the LT50 could be used as a reliable index for evaluating cold tolerance in chrysanthemum. At the same time, it was found that the cold tolerance of each chrysanthemum variety was relatively stable after gradually decreasing natural low-temperature exercise, and the evaluation result of cold tolerance was relatively reliable. Liu et al. (2014) determined the physiological indices of 18 *Spiraeas* varieties under low-temperature stress, obtained the membership function values of the physiological indices of cold tolerance and LT50s through REC, evaluated the cold tolerance in combination with field investigation, and screened out the varieties of *Spiraea* with strong cold tolerance. Li (2010) studied ‘Cold Purple’ and ‘Cold Yellow’ chrysanthemum flowers and found that the REC and LT50 were related to the tongue and tube flowers, and the REC significantly increased with decreasing temperature. The cold tolerance of the ‘Cold Yellow’ variety was stronger than that of ‘Cold Purple’, and the cold tolerance of the tube flowers of both varieties was stronger than that of the tongue flowers. In conclusion, REC can be used as an evaluation index of cold tolerance alone, or LT50 can be calculated by combining the logistic equation for evaluation. Both REC and LT50 are important indices for evaluating cold tolerance in chrysanthemum.

### 2.4 Anatomical structure

Plants growing in cold areas in nature have been in low-temperature environments for a long period of time, and their morphological and anatomical structures have undergone a series of adaptive changes to the environment, which gradually resulted in cold-resistant plants. The leaf epidermal structure includes the epidermis, cuticle, mesophyll, palisade tissue and spongy tissue. Many studies have found that the components of the leaf epidermal structure are closely related to cold tolerance (Zhao et al., 2005; Cui and Ma, 2007). In addition, Ai et al. (2006) found that the ultrastructure of cells was closely related to cold tolerance.

In terms of chrysanthemum cold tolerance, Xu et al. (2009) found that the LT50 of chrysanthemum leaves was significantly negatively correlated with the palisade tissue thickness, palisade tissue tightness, epidermal thickness, and palisade tissue/spongy tissue ratio and significantly positively correlated with palisade tissue porosity. Through path analysis, it was also found that the anatomical structure of leaves not only directly affected the cold tolerance of the chrysanthemum but also indirectly affected the change in the soluble sugar (SS) content. A mathematical model for comprehensive evaluation of the cold tolerance chrysanthemum was established by selecting the palisade tissue/spongy tissue ratio, palisade tissue tightness, palisade tissue porosity and SS content change rate as indices. Hu et al. (2000) found that after low-temperature treatment, the ultrastructure of the leaf organelles of a cut-flower chrysanthemum showed obvious changes, including the degradation of large starch grains, an increase in the number of mitochondria and lipoid particles, and slight disturbance of the lamellar structure. The organelles of varieties with strong cold tolerance were relatively stable. In conclusion, the structural composition of the leaf epidermis and ultrastructural changes in organelles can also be used as evaluation indices of cold tolerance in chrysanthemum.

There are relatively few studies on the phenotype of cold tolerance in chrysanthemum, mainly focusing on the aspects of the foot bud, florescence, REC, LT50 and leaf epidermal structure, among which the relationship between florescence and cold tolerance needs further investigation. In addition, research on evaluation indices that can directly evaluate chrysanthemum cold tolerance is insufficient, and it is necessary to strengthen research on the phenotype of cold tolerance in combination with new technologies and new theories to lay a foundation for future physiological and molecular studies in chrysanthemum.

## 3 Research advance on the physiological mechanisms of cold tolerance in chrysanthemum

Compared with the study of chrysanthemum phenotypic indices, the study of physiological mechanisms can more directly reflect the degree of low-temperature stress in plants. Many studies on the physiological mechanisms of cold tolerance in chrysanthemum have mainly focused on the biofilm system, protective enzyme system, osmotic regulatory substances, photosynthesis and hormones.

### 3.1 Biofilm system

Biofilms provide not only a relatively stable environment for cells but also a medium for transport, energy transfer and information exchange between cells and the outside world. Lyons (1970) believe that the cell membrane is the initial site damaged by low temperature, and its integrity and permeability are closely related to plant cold tolerance. To date, many studies of biofilms and cold tolerance have mainly focused on the changes in fatty acid, phospholipid and malondialdehyde (MDA) contents.

#### 3.1.1 Fatty acids

Studies on plant cold tolerance have found that fatty acids play an important role in biofilm structure (Li and Li, 2009), and the proportion and content of unsaturated fatty acids in membrane lipids are closely related to plant cold tolerance (Murata and Los, 1997). Under normal conditions, membrane lipids are in the liquid crystal phase, and at low temperature, plants maintain the state of membrane lipids by reducing the unsaturation of membrane fatty acids (Lyons et al., 1973; Li et al., 2019). Murata et al. (1992) believed that the higher the content of unsaturated fatty acids in membrane lipids is, the stronger the cold tolerance of plants is. Related studies in chrysanthemum have mainly concentrated on the contents and proportions of saturated and unsaturated fatty acids in leaves and roots. Li et al. (2013) found that the main membrane lipid fatty acids in the leaves and roots of the autumn chrysanthemum early flowering varieties ‘Taiping Xiaogu’ and ‘Jin Fengling’ and the late flowering varieties ‘Xingguang Canlan’ and ‘Mobao’ of autumn chrysanthemums mainly included palmitic acid (C16:0), linoleic acid (C18:2) and linolenic acid (C18:3). The main unsaturated fatty acids were C18:3 in the leaves and C18:2 in the roots, and the saturated fatty acid was C16:0. In addition, it was found that the fatty acid contents in leaves and roots showed different low-temperature response mechanisms, and there were obvious differences between the C18:3/oleic acid (C16:1) + C18:2 ratios in the leaves and the C18:2/C16:1 + C18:2 + C18:3 in the roots. The cold tolerance of C18:3 in leaves was greater than that of C18:2, while that of C18:2 in roots was greater than that of C18:3. The C18:3/C16:1 + C18:2 and C18:2/C16:1 + C18:2 + C18:3 ratios can be used as effective indicators to identify differences in the cold tolerance of leaves and roots between varieties. Wang et al. (2014) used nine autumn chrysanthemum varieties and found that the unsaturated fatty acid with the highest content in leaves was C18:3. The C18:3/C16:1 + C18:2 ratio significantly changed under low-temperature stress and could be used as an effective index to identify cold-tolerant autumn chrysanthemum varieties. In addition, through cluster analysis, the autumn chrysanthemum varieties were divided into three categories according to the strength of their cold tolerance, and the three varieties with strong cold tolerance were ‘Starlight Bright’, ‘Mobao’ and ‘Sandalwood Lion’, while according to their LT50 values, the varieties with strong cold tolerance were ‘Starlight Bright’, ‘Mobao’ and ‘Tongque Chunshen’.Comprehensive analysis identified the late flower varieties ‘Starlight Bright’ and ‘Mobao’ as having the strongest cold tolerance.

In conclusion, cold tolerance in chrysanthemum is related to the content of unsaturated fatty acids, and the main components of unsaturated fatty acids in leaves and roots are different: (C18:3) in leaves and C18:2 in roots.C18:3/C16:1 + C18:2 and C18:2/C16:1 + C18:2 + C18:3 can be used as important indicators to identify the differences in leaf and root cold tolerance between varieties. In addition, Li et al. (2013) and Wang et al. (2014) came to consistent research conclusions on leaves, which may be due to a significant overlap in the experimental materials. However, the cold-resistant varieties screened by Wang et al. (2014) using two different evaluation methods had certain differences, possibly due to the characteristics of the varieties themselves, and further in-depth research is needed.

#### 3.1.2 Phospholipids

Phospholipids are both functionaly and structurally active substances that have important physiological functions in biological activities, and certain amounts of phospholipids are present in all biological tissues (Yang, 2012). Plant biofilms are the first to be destroyed when plants are subjected to low temperature and freezing damage, and they play a key role in plant cold tolerance (Steponkus, 1984). Studies on cold tolerance in chrysanthemum have mainly focused on the contents of phospholipids in different parts and the response mechanism to low temperature. Yang (2012) found that the main component of phospholipids in leaves and roots was phosphatidylethanolamine (PE) in various chrysanthemum varieties, but its content was different and was generally higher in leaves than in roots. However, phosphatidylglycerol (PG), phosphatidylcholine (PC) and phosphatidylserine (PS) were not detected or were detected in trace amounts. In addition, the membrane component contents in leaves and roots of different chrysanthemum varieties had different response mechanisms to low temperature. The PE content in the leaves of most chrysanthemum varieties increased with decreasing temperature, while that in the roots increased, and the increase in the PE content contributed to the increase in the unsaturated fatty acid content in the phospholipid membrane. In conclusion, PE in phospholipid components has a certain relationship with cold tolerance. Under low-temperature stress, the PE content in leaves increases, while the PE content in roots varies, and the higher the PE content is, the higher unsaturated fatty acid content is, which indirectly indicates that the cold tolerance of leaves is stronger than that of roots.

#### 3.1.3 Malondialdehyde

During stress, plants produce excess free radicals, which trigger or aggravate membrane lipid peroxidation. Both intermediate free radicals and MDA, the final product of membrane lipid peroxidation, can seriously damage biofilms (Chen, 1991; Yang, 2018). Low-temperature stress can cause changes in the permeability of the plant cell membrane, with the semipermeability of the cell membrane reduced or lost, and increase the content of MDA. The change in the MDA content can reflect the degree of cell membrane disturbance. Xu et al. (2010) found that with decreasing temperature, the MDA content in the leaves of five groundcover chrysanthemum strains increased to varying degrees, and lipid peroxidation in the cell membrane continuously increased, resulting in a sudden increase in electrolyte exosmosis due to cell membrane damage. Li (2010) studied cold tolerance in the ‘Cold Purple’ and ‘Cold Yellow’ varieties and found that the MDA content was closely related to the tongue and tube flowers of ‘Cold Purple’. When the temperature was lower than 6°C, the MDA content in the tongue flowers of ‘Cold Purple’ continuously increased. In a study of ‘Jinlong Tengyun’ chrysanthemum, Cheng et al. (2018) also found that the overall MDA content in leaves increased with decreasing temperature. Li et al. (2011) found that in a low-temperature environment, the protective enzyme system was deactivated, and the MDA content first decreased and then increased. Li et al. (2009) found that the MDA content in the leaves of autumn white chrysanthemum after low-temperature exercise was lower than that in the control group, which could weaken the peroxidation of membrane lipids and promote cold tolerance.

As shown in **Table 1**, the MDA content of different chrysanthemum varieties increased overall with decreasing temperature. Once the MDA content increased, the degree of lipid peroxidation of the cell membrane intensified, the plant cell membrane system began to be persecuted, and the membrane system lost its original selective permeability, resulting in electrolyte leakage and a decrease in cellular water potential, and the cold tolerance gradually weakened. This change is irreversible. According to Li’s research, the MDA content of chrysanthemums undergoing low-temperature exercise is lower than that of chrysanthemums without low-temperature exercise, which can weaken the peroxide effect and reduce the MDA content. However, the time of low-temperature exercise and low-temperature stress is relatively short, which can further explore the relationship between the duration of low-temperature exercise and MDA content.

**Table 1:** Changes in MDA content in different chrysanthemum varieties under low-temperature.

Chrysanthemum variety	Temperature range	Change trend	Reference
Ground-cover chrysanthemum	-5°C~14°C	Increase	Xu et al., 2010
‘Cold yellow’ ‘Cold Purple’	3°C~9°C 3°C~9°C	Increase Increase	Li, 2010 Li, 2010
‘Jinlong Tengyun’	-8°C~16°C	Increase	Cheng et al., 2018
*Gazania rigens*	-6°C~6°C	Initial decrease followed by an increase	Li et al., 2011
Autumn chrysanthemum	-5°C~4°C	Initial decrease followed by an increase and then a decrease	Li et al., 2009

### 3.2 Protective enzyme system

Reactive oxygen species (ROS) produced by plant metabolism mainly include oxygen-containing substances such as hydrogen peroxide (H_2_O_2_), superoxide anion (O^2-^) and hydroxyl radical (-OH) (Eva et al., 2002; Apel and Hirt, 2004). Plants produce more ROS under low-temperature stress, and a large amount of ROS accumulation can inactivate proteases and increase peroxidation of membrane lipids, causing serious damage to cells (Wang et al., 1986; Green and Fluhr, 1995; Dat et al., 2000; Hu et al., 2009; Xu et al., 2020). The protective enzyme system to remove ROS mainly consists of superoxide dismutase (SOD), peroxidase (POD), catalase (CAT), ascorbate peroxidase (APX), glutathione reductase (GR) and other components (Li et al., 2021). Among them, SOD, POD and CAT are more sensitive to low-temperature stress and are often used as important indicators to characterize plant cold tolerance of plants (Hao et al., 2014; Jiang et al., 2014).

#### 3.2.1 Superoxide dismutase

The main function of SOD is to clear O^2-^ and catalyse the reaction of O^2-^ and H^+^ to produce H_2_O. Under the stress of low temperature (Kayihan et al., 2012), the SOD enzyme activity of plants can increase. The increase in SOD activity can improve the cold tolerance of plants, but its effect is limited, and SOD cannot play a normal role under severe low-temperature stress. In the study of cold tolerance and SOD in chrysanthemum, the main focus has been on the change in SOD activity. Xu et al. (2010) found that the SOD activity in the leaves of five groundcover chrysanthemum strains increased with decreasing temperature, and the accumulation of SOD promoted cold tolerance in these strains. However, with the continuous decrease in temperature, the SOD activity decreased but was still higher than the initial SOD activity, indicating that the groundcover chrysanthemum strains had a strong ability to adapt to low-temperature stress. Li (2010) found that when the temperature was not lower than 6°C, the cold tolerance of the ‘Cold Yellow’ and ‘Cold Purple’ varieties was improved by adjusting SOD activity. When the temperature was further reduced, their own adjustment ability was weakened, resulting in low-temperature damage, and overall, the adjustment ability of ‘Cold Yellow’ was stronger than that of ‘Cold Purple’. Cheng et al. (2018) found that under low-temperature stress, the SOD activity in the leaves of the chrysanthemum ‘Jinlong Tengyun’ showed an overall trend of first increased and then decreased with decreasing temperature, possibly due to low-temperature damage. Li et al. (2009) found that after low-temperature exercise, autumn white chrysanthemum still had higher SOD activity under low-temperature stress than that without low-temperature exercise. Bai et al. (2013) measured the SOD activity in overwintering leaves and foot buds of ‘Purple Medal’ in an open field and found that in the leaves, SOD activity first increased and then decreased with decreasing temperature, while the SOD activity in the foot buds first increased, then decreased and then increased again, indicating that the foot buds had strong adaptability to a low-temperature environment and stronger cold tolerance than the leaves.

In conclusion, SOD activity increased gradually overall with the decreasing temperature, but when the temperature exceeded the capacity of chrysanthemum plants, SOD activity decreased, and the plants began to suffer from low-temperature persecution. In addition, the cold tolerance of different chrysanthemum varieties is different, cold tolerance in different parts of the same plant is different, and the cold tolerance of the foot bud is stronger than that of the leaf.

#### 3.2.2 Peroxidase

The main function of POD is to clear H_2_O_2_ in plants, generate H_2_O and O_2_, and reduce damage to plants (Chaoui et al.,1997). Under abiotic stress conditions such as low temperature (Zhang and Xu, 2009), POD activity increases, which helps improve plant tolerance to low temperature. However, when the low temperature exceeds the critical temperature and the ROS generation rate far exceeds the clearance capacity of POD, the POD activity decreases. Studies on chrysanthemum cold tolerance have mainly focused on the change in POD activity in response to temperature. Cheng et al. (2018) found that the POD activity in the leaves of the chrysanthemum ‘Jinlong Tengyun’ first increased and then decreased with decreasing temperature. Li et al. (2009) found that under -5°C stress, the POD activity in autumn chrysanthemum after low-temperature exercise was consistent with that in the control group, an initial increase followed by a decrease, and the POD activity after low-temperature exercise was stronger. Bai et al. (2013) determined the POD activity in the leaves and foot buds of ‘Purple Medal’ overwintering in an open field and found that the POD activity in both parts first decreased and then increased with decreasing temperature. However, starting with the flower organs, Li (2010) found that the changes in POD activity under low-temperature stress had a poor correlation with chrysanthemum varieties, and their cold tolerance at different flowering stages could not be judged, which was not suitable for the study of the tolerance indices of ‘Cold Yellow’ and ‘Cold Purple’ chrysanthemum. In conclusion, with decrease temperature, POD activity increased overall, but when the temperature exceeded the temperature tolerance range of the chrysanthemum plants, POD activity decreased, and ROS and H_2_O_2_ began to accumulate, causing stress. In addition, the cold tolerance of different varieties of chrysanthemum is different, and the cold tolerance of flower organs of some chrysanthemum varieties is not suitable for studying changes in POD activity.

#### 3.2.3 Catalase

CAT is a key enzyme in the removal of H_2_O_2_ in plants (Willekens et al., 1995), and its main function is to catalyze H_2_O_2_ to produce H_2_O and O_2_. CAT has a high enzyme activity rate and can efficiently remove H_2_O_2_, but it has a weak affinity for H_2_O_2_ and mainly removes high concentrations of H_2_O_2_ (Lu, 2007). Studies on cold tolerance chrysanthemum have mainly focused on changes in POD activity. Cheng et al. (2018) found that under low-temperature stress, CAT activity in the leaves of ‘Jinlong Tengyun’ chrysanthemum first increased and then decreased with decreasing temperature. Bai et al. (2013) measured the CAT activity in overwintering leaves and foot buds of ‘Purple Medal’ in an open field and found that the changes in CAT activity in the different parts were different. With the decrease in temperature, the CAT activity in the leaves first rised and then decreased, and that in the foot buds first increased, then decreased and then increased again. In conclusion, with decreasing temperature, CAT activity gradually increased overall, but when CAT activity reached an extreme value, the scavenging ability of CAT on H_2_O_2_ was the strongest, and the concentration of H_2_O_2_ began to decline, and the activity of CAT also declined. In addition, the cold tolerance of different varieties of chrysanthemum is different, the cold tolerance of different the same is different, and the cold tolerance of the foot bud is stronger than that of the leaf.

In addition, there are other enzymes that contribute to the response of chrysanthemum to low-temperature stress. Li et al. (2009) found that with increasing low-temperature stress time, the 5’-nucleotidase activity in chrysanthemum gradually decreased but overall, was significantly higher after low-temperature exercise than that in the control group, which could maintain the stability of the cell membrane and indirectly improve cold tolerance in chrysanthemum.

**Table 2** shows that SOD, POD and CAT have a certain synergistic effect, and also have a certain temperature adjustment range. Under mild low-temperature stress, chrysanthemum plants produced a stress response, the activity of antioxidant enzymes increased, and the ROS scavenging ability improved, which can alleviate low-temperature injury. With the decrease in temperature, the activity of antioxidant enzymes in plants began to decline, and the ability of plants to alleviate the damage from ROS became weak. As the temperature continued to decrease, it exceeded the ability of chrysanthemum to adjust. As its own adjustment ability was limited, the enzyme activity was significantly reduced, resulting in severely damaged from low temperature. In particular, the enzyme activity of CAT decreased within the temperature range of the chrysanthemum itself; this is related to the characteristics of CAT, which mainly removes high concentrations of H_2_O_2_. When H_2_O_2_ is jointly removed by highly active POD and CAT, the concentration of H_2_O_2_ decreases, and the activity of CAT may also decrease.

**Table 2:** Changes in the protective enzyme activity of different chrysanthemum varieties under low-temperature.

Protective enzyme	Chrysanthemum variety	Temperature range	Change trend	Reference
SOD	Ground-cover chrysanthemum	-5°C~14°C	Initial increase followed by a decrease	Xu et al., 2010
	‘Cold yellow’ ‘Cold Purple’	3°C~6°C 3°C~6°C	Initial increase followed by a decrease Initial increase followed by a decrease	Li, 2010 Li, 2010
	‘Jinlong Tengyun’	-8°C~16°C	Initial increase followed by a decrease	Cheng et al., 2018
	Autumn chrysanthemum	-5°C~4°C	Initial increase followed by a decrease	Li et al., 2009
	‘Purple Medal’	Natural wintering	Initial increase followed by a decrease (Leaf)	Bai et al., 2013
			Initial increase followed by a decrease and then an increase (Foot bud)	
POD	‘Jinlong Tengyun’	-8°C~16°C	Initial decrease followed by an increase and then a decrease	Cheng et al., 2018
	Autumn chrysanthemum	-5°C~4°C	Initial increase followed by a decrease	Li et al., 2009
	‘Purple Medal’	Natural wintering	Initial decrease followed by an increase	Bai et al., 2013
CAT	‘Jinlong Tengyun’	-8°C~16°C	Initial increase followed by a decrease	Cheng et al., 2018
	‘Purple Medal’	Natural wintering	Initial increase followed by a decrease (Leaf)	Bai et al., 2013
			Initial increase followed by a decrease and then an increase (Foot bud)	

### 3.3 Osmoregulatory substances

Under low-temperature stress, plants can regulate cell osmotic pressure by producing and accumulating osmotic regulatory substances to avoid significant loss of cell water; this can alleviate the damage to plants caused by low temperature to a certain extent (Gao et al., 2016). Soluble sugar (SS), soluble protein (SP) and proline (Pro) are important osmoregulatory substances in plants, and SP content can reflect the response of plants to low temperature.

#### 3.3.1 Soluble sugar

SS are not only important energy sources for plant respiration but also play a role in regulating cell osmotic pressure (Morsy et al., 2007), which can increase the concentration of cell fluid and increase cell water retention and nonfrozen water in tissues. The SS content is positively correlated with the cold tolerance of plants (Wang, 1987). Zheng et al. (1994) showed that some winter chrysanthemum varieties could withstand a low temperature of -20°C, and their cold tolerance was better than that of autumn chrysanthemum varieties because of their lower leaf water content and higher SS content. Mao et al. (2004) studied cold tolerance in the groundcover chrysanthemums ‘Xiaruahuang’ and ‘9871’ and found that the water content of the plants decreased and the cold and freezing tolerance increased. There was a negative correlation between the water content and SS content in the chrysanthemum plants, and the relationship was more obvious when the temperature decreased. Xu et al. (2010) studied the cold tolerance of five groundcover chrysanthemum strains and found that with the decrease in temperature, the SS content began to increase the changes caused by low temperature. When the temperature dropped to a certain extent, the sugar contents of strains 074-17, 074-123, 074-237, and 074-428 stopped increasing and began to decrease, indicating that the groundcover chrysanthemums had a certain tolerance limit to low temperature, and the increase in the SS content also had a certain temperature range limit. Li (2010) found that with the decrease in temperature, ‘Cold Yellow’ had a stronger ability to enhance its cold tolerance than ‘Cold Purple’ by accumulating SS, and the cold tolerance of tubular flowers of the same variety was stronger than that of tongue flowers. Chen et al. (2018) studied cold tolerance in perennial chamomile and found that the SS content first increased and then decreased with decreasing temperature as a stress response, which could be used for the determination of cold tolerance, and the cold tolerance of perennial chamomile was good near 0°C. Cheng et al. (2018) found that the SS content in the leaves of the autumn chrysanthemum ‘Jinlong Tengyun’ first increased and then decreased with the extension of the low-temperature treatment time and the decrease in temperature, indicating that chrysanthemum cold tolerance could be improved through an increase in the SS content under mild low-temperature stress. However, with the continuous decrease in temperature, low-temperature stress prevented SS synthesis. They were even hydrolysed, further reducing the content. Bai et al. (2013) observed a ‘Purple Medal’ natural low-temperature strip and found that the SS content in the leaves significantly increased with decreasing temperature, while that in foot buds first increased, then decreased and then increased again.

In conclusion, the change in the SS content was positively correlated with cold tolerance. With the decrease in temperature, the contents of different SS increased overall, but when the temperature exceeded a certain range, SS synthesis is blocked, and the content began to decline. In addition, cold tolerance in different parts of the same plant was different, and tubular flowers had stronger tolerance than tongue flowers. The foot buds had stronger tolerance than leaves.

#### 3.3.2 Soluble protein

SP, similar to SS, are nutrients in plant cells. Most of them are enzymes involved in various reactions; SP can also regulate cell osmotic pressure, and the increase in their accumulation can effectively prevent plant dehydration. The hydrophilic colloids of SP are strong, and an increase in their content can significantly enhance water retention by cells. Proteins also regulate gene expression during cold stress, resulting in improveed cold tolerance. Xu et al. (2010) studied cold tolerance in five strains of groundcover chrysanthemum and found that with decreasing temperature, the SP content increased, the cell fluid concentration was adjusted, and tolerance to low-temperature stress was observed. Li (2010) found that the SP contents of ‘Cold Yellow’ and ‘Cold Purple’ chrysanthemums first increased and then decreased with decreasing temperature. The ability of ‘Cold Purple’ to resist low-temperature stress through SP content adjustment was weaker than that of ‘Cold Yellow’, and ‘Cold Purple’ was more sensitive to low temperature; the cold tolerance of the tube flowers of the two varieties was stronger than that of the tongue flowers. Li et al. (2011) found that related protein synthesis genes were switched on at -3°C in *Gazania rigens*, thus increasing the cell protein concentration and decreasing the freezing point to resist low temperature. However, at -3°C to -6°C, the protein content dropped sharply, beyond the range of self-regulation. Chen et al. (2018) found that the SP content of *Gazania rigens* first increased and then decreased under low-temperature stress, which could be used for the determination of cold tolerance. Bai et al. (2013) determined the SP content in the leaves and foot buds of ‘Purple Medal’ overwintering in an open field and found that the SP content in both leaves and foot buds first decreased, then increased and then decreased again. In conclusion, with the decrease in temperature, the contents of different varieties increased overall, but when the temperature exceeded a certain range, the SP content began to decline. In addition, the cold tolerance of different parts of the same plant was different, and tubular flowers had stronger tolerance than tongue flowers.

#### 3.3.3 Proline

Pro is an important osmoregulatory substance in plant cells, which has strong hydration ability and can prevent cell dehydration under stress (Yao et al., 2000). Pro can also increase the hydrophilicity of proteins and maintain the conformation of enzymes at low temperatures (Jiang et al., 2002). The Pro content significantly increases under low-temperature stress, and its main function is to maintain cell water potential and enhance cell water retention under stress. Xu et al. (2010) studied the cold tolerance in five groundcover chrysanthemum strains and found that the free Pro content increased in the early stage of low-temperature stress, but as the temperature continued to drop, the free Pro content in the leaves of each strain decreased; as the temperature continued to decrease, the Pro content began to rapidly increase, indicating that it was sensitive to temperature stress. Thus, Pro content plays an important role in regulating cold tolerance in the groundcover chrysanthemums. Li et al. (2011) found that with the increasing intensity of low-temperature stress, the Pro content in the leaves of *Gazania rigens* first increased and then decreased. Li et al. (2009) found that after low-temperature exercise, the Pro content in autumn white chrysanthemum first increased and then decreased and was significantly higher than that in the control group. Bai et al. (2013) found that the Pro content in leaves and foot buds of ‘Purple Medal’ overwintering in an open field first decreased, then increased and then decreased again. In conclusion, with the decrease in temperature, the Pro content of different varieties increased overall, but when the temperature exceeded a certain range, the Pro contents began to decline. The Pro content of the ground cover first increased and then decreased with decreasing temperature, which is possibly related to the strong water absorption ability of the ground cover. In addition, the variation trends of SP and Pro in some chrysanthemum varieties were roughly the same, indicating that there was a certain relationship between them, and some proteins may regulate the expression of Pro-related genes.

**Table 3** shows that with decreasing temperature, SS, SP and Pro contents showed a certain correlation. Among them, the contents of SS and Pro showed more similar trends, which is possibly related to the relatively similar functions of SP and Pro. In addition, SP may indirectly regulate the expression of Pro biosynthesis-related genes and others, which needs to be further studied in combination with new technologies. As the intensity of external low-temperature stress increases and the stress time continues to exceed the temperature tolerance range of chrysanthemum, the related tissues and organs are damaged and unable to produce more SS, SP and Pro, thus reducing the content of osmoregulatory substances.

**Table 3:** Changes in osmotic regulator content in different chrysanthemum varieties under low-temperature.

Osmotic regulator	Chrysanthemum variety	Temperature range	Change trend	Reference
SS	Ground-cover chrysanthemum	-5°C~14°C	Initial increase followed by a decrease	Xu et al., 2010
	‘Cold yellow’ ‘Cold Purple’	3°C~12°C 3°C~12°C	Increase Increase	Li, 2010
	‘Jinlong Tengyun’	-8°C~16°C	Initial increase followed by a decrease	Cheng et al., 2018
	*Perennial chamomile*	-10°C~5°C	Initial increase followed by a decrease	Chen et al., 2018
	‘Purple Medal’	Natural wintering	Initial increase followed by a decrease	Bai et al., 2013
SP	Ground-cover chrysanthemum	-5°C~14°C	Increase	Xu et al., 2010
	‘Cold yellow’ ‘Cold Purple’	3°C~12°C 3°C~12°C	Initial increase followed by a decrease Initial increase followed by a decrease	Li, 2010 Li, 2010
	*Gazania rigens*	-6°C~6°C	Initial increase followed by a decrease	Li et al., 2011
	*Perennial chamomile*	-10°C~5°C	Initial increase followed by a decrease	Chen et al., 2018
	‘Purple Medal’	Natural wintering	Initial decrease followed by an increase and then a decrease	Bai et al., 2013
Pro	Ground-cover chrysanthemum	-5°C~14°C	Initial increase followed by a decrease and then an increase	Xu et al., 2010
	*Gazania rigens*	-6°C~6°C	Initial increase followed by a decrease	Li et al., 2011
	Autumn chrysanthemum	-5°C~4°C	Initial increase followed by a decrease	Li et al., 2009
	‘Purple Medal’	Natural wintering	Initial decrease followed by an increase and then a decrease	Bai et al., 2013

### 3.4 Photosynthesis

As the most important metabolic process in plants, photosynthesis refers to the process during which plants absorb light energy and convert it into stable chemical energy, assimilate CO_2_ and H_2_O, produce organic matter and release O_2_ (Li et al., 2012), which is closely related to external environmental factors. Many studies have found that the photosynthetic rate of plants significantly decreases under low-temperature conditions (Rapacz et al., 2001; Guo et al., 2009; Yang et al., 2012). To date, many studies on photosynthesis and cold tolerance in chrysanthemum have mainly focused on the photosystem, chlorophyll content and thylakoid membrane lipid structure.

#### 3.4.1 Photosystem

Low-temperature stress can damage plant photosystem II (PSII) and photosystem I (PSI), hinder photosynthetic electron transport, and generate excess light energy to destroy the photosynthetic reaction centre (Liu et al., 2001; Liu et al., 2009). Studies on the cold tolerance and photosynthetic system of chrysanthemum have mainly focused on the effects of changes in initial fluorescence (F_0_), maximum photochemical efficiency (F_v_/F_m_), potential photochemical efficiency (F_v_/F_0_), photosynthetic electron transport quantum efficiency (Φ), photochemical quenching coefficient (Q_P_) and nonphotochemical quenching coefficient (N_P,Q_) on PSII and PSI under low-temperature stress.

To date, most studies on the chrysanthemum photosystem have only considered the photochemical reaction of PSII and rarely involved PSI. Liang et al. (2010) found that under low-temperature and low-light treatment, F_0_ significantly increased, F_v_/F_m_ significantly decreased, and Φ_PSII_, Q_P_ and the apparent photosynthetic electron transfer rate decreased with increasing stress degree and stress time, which could be used as an evaluation index of chrysanthemum tolerance to low temperature and low light. Cheng et al. (2018) found that under 4°C treatment, F_v_/F_m_ and Q_P_ of the autumn chrysanthemum ‘Jinlong Tengyun’ did not significantly change, while N_P,Q_ increased, indicating that under short-term low-temperature stress, the PSII function of the chrysanthemum was affected and the chrysanthemum consumed excess excitation energy by increasing heat dissipation to protect the photosystem from damage. At -8°C, F_v_/F_m_ and Q_P_ both significantly decreased, and N_P,Q_ was almost 0, indicating that the photosynthetic system in ‘Jinlong Tengyun’ leaves was seriously damaged and that the photosynthetic capacity was essentially lost. Nie et al. (2020) also studied the autumn chrysanthemum ‘Jinlong Tengyun’ and found that at -4°C, F_v_/F_m_, F_v_/F_0_ and Q_p_ declined, while N_P,Q_ first decreased and then increased, indicating that the chrysanthemum leaves responded to low-temperature stress by reducing PSII light energy utilization and photochemical efficiency and increasing heat loss. These results were consistent with the findings of Cheng et al. (2018), but the photosynthetic system was not seriously damaged in this study.

The photochemical reaction is a collaborative result of PSII and PSI, and the continuous excitation rapid chlorophyll fluorescence technique can accurately detect PSII and PSI related changes in plants (Li et al., 2005; Gao et al., 2013; Qi et al., 2015). Zhao et al. (2019) used this technology to study the cut-flower chrysanthemum ‘White Ping-Pong’ and found that under -4°C low-temperature stress, the openness of the photosystem reaction centre was reduced, and some reaction centres were inactivated, which caused serious damage to PSI and reduced the overall photosynthetic activity of the plant. Zhao (2019) conducted the same research on the chrysanthemum ‘Tangyu Jinqiu’ and found that the coordination between PSII and PSI was weakened at low temperature, the fluency of energy transfer was reduced, the energy utilization rate of PSI was decreased, PSI was seriously damaged, and the overall performance of the photosynthetic mechanism of the chrysanthemum leaves decreased at low temperature. The continuous excitation rapid chlorophyll fluorescence technique was not conducive to photochemical reactions and the accumulation of organic matter.

In summary, cold tolerance in chrysanthemum is mainly related to functional changes in the light system. Low-temperature stress causes changes in various photosynthetic indices and then affects the light system. The light system is protected from damage by increasing heat dissipation, but this protection has a certain temperature range beyond which serious damage occurs. In addition, continuous excitation rapid chlorophyll fluorescence technology allows quick and accurate study of changes in the chrysanthemum photosystem under low-temperature stress.

#### 3.4.2 Chlorophyll

Under low-temperature stress, the decrease in plant photosynthesis is often related to changes in photosynthetic pigments. Chlorophyll is the main component of photosynthetic pigments in plants and is responsible for capturing and transferring light energy in the process of photosynthesis. In the study of cold tolerance in chrysanthemum, changes in the chlorophyll content may be different in response to different low-temperature stresses. Nie et al. (2020) found that under short-term low-temperature stress, the chlorophyll a/chlorophyll b ratio in chrysanthemum increased with decreasing temperature. Although the photosynthetic organs of PSII in plants were affected under low-temperature stress, the reaction centre was relatively stable, and therefore, it was inferred that chlorophyll a was more distributed in the PSII reaction centre and chlorophyll b was more distributed in the light-collecting pigment protein complex (Wang et al., 2003; Wang J. et al., 2006; Lichtenthaler and Buschaann, 2001; Ebrahimiyan et al., 2013). Cheng et al. (2018) found that the contents of chlorophyll a, chlorophyll b and chlorophyll a + b in chrysanthemum leaves decreased trend with the extension of the low-temperature stress time and the decrease in temperature. The chlorophyll content of the chrysanthemum and the ability to capture and use light energy decreased, and the destruction of the photosynthetic structure was avoided, which was conducive to the adaptation of the chrysanthemum to low-temperature stress. In conclusion, chrysanthemums can cope with low-temperature stress through changes in chlorophyll content. Under short-term low-temperature stress, chlorophyll content increased to ensure the stability of the light reaction centre. Under long-term low temperature stress, chlorophyll content decreased to avoid the destruction of photosynthetic structure.

#### 3.4.3 Thylakoids

The light reaction of photosynthesis is carried out on the thylakoid membrane, which has a high fluidity. Relevant studies have shown that low temperature can affect plant photosynthesis by affecting the structure of the thylakoid membrane (Dai et al., 2004). In addition, the photochemical reaction is the result of coordination between PSII and PSI, and improving the efficiency of PSII and PSI at the same time can enable efficient photosynthesis (Yao et al., 2009). In a study on the cold tolerance of chrysanthemum, Zhao (2019) found that the main fatty acids in the leaf thylakoid membrane were C16:0, followed by stearic acid (C18:0) and C16:1, and the contents of C16:0 and C18:0 gradually decreased with decreasing temperature under low-temperature stress, while that of C16:1 first decreased and then increased with decreasing temperature. The proportions of C18:2 and C18:3 increased with decreasing stress temperature. In addition, Zhao et al. (2019) found in their study on the cut-flower chrysanthemum variety ‘White Ping-Pong’ that the light-trapping pigment in leaves was degraded under low-temperature stress, resulting in reduced openness and partial deactivation of the PSII reaction centre, damage to the oxygen-releasing complex, and indirectly reduced activity of the PSI reaction centre. In addition, the PSI of cut chrysanthemum leaves was seriously damaged at -4°C, which blocked the energy transfer between thylakoids, reduced the protection ability of the photosynthetic system, and decreased the overall photosynthetic performance.

In summary, studies on the cold tolerance of thylakoids and chrysanthemums have mainly focused on the effect of changes in the unsaturated fatty acid content in membrane lipids on the structure of thylakoid membranes and the effect of degradation of light-capturing pigments in the light system on the light response. Cold tolerance in chrysanthemum can be judged by changes in the composition of the thylakoid membrane and the light response performance.

### 3.5 Hormones

To help plants cope with stress, exogenous hormones can be sprayed onto plants, and the content of endogenous hormones can be adjusted to cope with low-temperature stress. To resist low-temperature stress, plants maintain high endogenous abscisic acid (ABA) levels. Many studies on cold tolerance and hormones in chrysanthemum have been started by applying exogenous hormones such as salicylic acid (SA), acetylsalicylic acid (ASA) and 5-aminolevulinic acid (ALA).

#### 3.5.1 Endogenous hormone ABA

ABA can inhibit cell division and elongation, thus hindering plant growth and promoting the formation of dormant buds to adapt to low-temperature stress (Thomashow, 1999; Jeon et al., 2010; Shi et al., 2012). Many studies have also confirmed that the endogenous ABA content of many plants significantly increased after low-temperature treatment and was higher in varieties with strong cold tolerance than in those with cold sensitivity (Chen et al., 2015). Zhang (2013) found that treatment of chrysanthemum with a low concentration of exogenous ABA could improve the antioxidant activity of ‘Shenma’ cut chrysanthemum cells under low-temperature conditions, reduce the content of ROS, reduce the damage caused by membrane lipid peroxidation, and enhance the osmotic regulation ability of cells, thus improving tolerance to low-temperature stress. ABA treatment also affected photosynthesis and dry matter accumulation in plants to a certain extent. In conclusion, the activities of antioxidant enzymes can be affected, the osmotic regulation ability can be enhanced, and cold tolerance in chrysanthemum can be indirectly improved through increases in the content of the endogenous hormone ABA.

#### 3.5.2 Exogenous hormones SA, ASA and ALA

SA is a small-molecule phenolic substance that widely exists in plants and can significantly reduce the membrane permeability of plant cells under low-temperature stress and improve the cold tolerance of plants (Wang et al., 2002; Cai et al., 2007). At present, the application of exogenous hormones to improve the cold tolerance of plants is an important approach to extending the ornamental period and improving the ornamental value of chrysanthemums. Li Y. H. et al. (2010) used the autumn chrysanthemum early flower variety ‘Tangyu Jinqiu’ and late flower variety ‘Xiangyun’ as test materials and found that after application of 1.0 mmol/L SA, the Pro content, net photosynthetic rate, SOD activity and CAT activity in leaves were higher at all temperatures than those in the control group sprayed with distilled water, while the MDA content was lower than that in the control group. SA can significantly improve cold tolerance in chrysanthemum. Tian (2009) also studied ‘Tangyu Jinqiu’, an early flower variety of autumn chrysanthemum, and ‘Xiangyun’, a late flower variety, and found that external application of SA could alleviate the effects of low temperature by adjusting the REC and chlorophyll, MDA, SS and Pro contents, thus improving cold tolerance.

ASA is a derivative of SA. Yang and Zheng (2018) used ‘Shenma’ cut-flower chrysanthemum and found that exogenous ASA, CaCl_2_ and ASA + CaCl_2_ could significantly increase the activities of SOD, APX, and GR in chrysanthemum leaves. In addition, by improving the metabolic activity of the ASA-GSH circulatory system, the damage caused by ROS to chrysanthemum seedlings could be reduced, and the tolerance of chrysanthemum to low temperature and low light could be improved. Moreover, the combination treatment with ASA and CaCl_2_ was better than that of ASA or CaCl_2_ alone.

ALA is a synthetic precursor of all porphyrins (chlorophyll, haem, photochromes, etc.) (Wang et al., 2005). Studies have found that low concentrations of ALA can regulate plant growth and development, promote crop yields and improve stress tolerance (Wang et al., 2003). In a study on the cold tolerance in chrysanthemum, Zhang et al. (2014) found that spraying 50 mg/L ALA on the leaf surface of ‘Cold white’ could significantly reduce the REC and MDA content, increase the chlorophyll, SS, and SP contents and SOD activity, and significantly increase the net photosynthetic rate, stomatal conductivity and transpiration rate in leaves. The photosynthetic capacity and cold tolerance of the cut-flower chrysanthemum were improved.

In conclusion, exogenous hormones sprayed on chrysanthemum can significantly improve physiological indices related to cold tolerance, such as the chlorophyll, MDA, SS, and Pro contents, REC, and SOD activity, and indirectly improve cold tolerance in chrysanthemum.

There are relatively few studies on the physiological mechanism of cold tolerance in chrysanthemums, mainly focusing on the biofilm system, protective enzyme system, osmotic regulatory substances, photosynthesis and hormones. However, there are relatively few studies on hormones and cold tolerance, and further analysis is needed on the effects of more endogenous hormones and exogenous hormones on cold tolerance. However, the correlation between physiological indicators and cold tolerance is not sufficiently clear, and it is necessary to combine genomics, transcriptomics, proteomics and other omics with new technologies to further study and analyse the physiological mechanisms of cold tolerance in chrysanthemum and, at the same time, lay the foundation for chrysanthemum molecular markers and breeding selection in the future.

## 4 Research advance on the forward genetics of cold tolerance in chrysanthemum

Compared with physiological indices, forward genetics studies can elucidate the genetic status of chrysanthemum cold tolerance. Many forward genetics studies on cold tolerance in chrysanthemums have mainly focused on genetic analysis, molecular markers, QTL mapping and genome-wide association analysis.

### 4.1 Genetic analysis

Chrysanthemums are crosspollinated plants; their genotypes are highly heterozygous, and their trait inheritance is extremely complex. In the early stage, genetic research on chrysanthemum characters was mainly conducted by investigating the separation ratios of characters in hybrid progeny populations and summarizing the genetic rules. Genetic analysis studies of chrysanthemum cold tolerance have mainly focused on the progeny separation ratio and mixed genetic model analysis. Zhang XJ et al. (2011) crossed a strain of the groundcover chrysanthemum ‘Qiuyan’, which has the strong cold tolerance *AtDREB1A* gene, as the maternal parent with the groundcover chrysanthemum ‘Ya dong Zhiguang’, which has excellent ornamental properties, and obtained 158 hybrid offspring. Among them, 55 hybrid offspring were examined by PCR and RT-PCR. There were 37 strains carrying and 18 strains not carrying the exogenous *DREB1A* gene, the ratio was approximately 2:1, indicating that the exogenous *AtDREB1A* gene could be stably inherited by offspring during sexual reproduction and that the offspring had strong cold tolerance.

The hybrid genetic model can be used to study the genetic mechanism of chrysanthemum through direct and indirect analyses. Chi et al. (2018) used the interspecific F_1_ hybrid between the brain diploid-related species of intolerant *Chrysanthemum* and hardy *Chrysanthemum* as materials. They found that three traits were closely related to cold tolerance, namely the LT50, the number of foot buds and the height of foot buds, which had a large variation range, with coefficients of variation between 21.0% and 51.80%, and there was a certain degree of heterosis and superparent isolated individuals. In addition, genetic control mechanisms of the three cold tolerance traits were identified by the mixed genetic model as follows: the LT50 was controlled by two pairs of additive-dominant-epistatic major genes, the number of foot buds was controlled by one pair of additive-dominant major genes, and the foot buds was controlled by one pair of additive-dominant genes. Ma (2017) used 100 F_1_ hybrid lines between strongly cold-tolerant ‘Nannong Xuefeng’ as the maternal parent and poorly cold-tolerant ‘Mengbai’ as the paternal parent and adopted the major gene plus multigene analysis method of the mixed genetic model of plant quantitative traits. The study found that chrysanthemum cold tolerance at the seedling and full flowering stages was mainly controlled by two pairs of major genes, which were manifested as additive, dominant and upper. However, no major gene controlling cold tolerance was found at the bud and foot bud stages, indicating that the genetic mechanisms of cold tolerance at different growth stages greatly differed. In addition, Ma found that the coefficient of variation in the cold tolerance of F_1_ generation tongue flowers was 43.60%, and the variation range exceeded those of both parents, with positive and negative hyperparental separation, which provided a research basis for the selection of cold-tolerant hyperparental strains and the improvement of cold-tolerant varieties.

In conclusion, recent genetic analyses of cold tolerance are mainly based on the separation ratio of recrossed offspring and on the analysis of genetic mechanisms with the help of a mixed genetic model. Using the mixed genetic model, Chi et al. (2018) indirectly analysed the genetic mechanism of cold tolerance using three traits, the LT50, the number of foot buds and the height of foot buds, which are closely related to cold tolerance. Ma (2017) directly used the major gene plus multigene method to analyse the genetic mechanism of cold tolerance and provided some references for the genetic analysis of cold tolerance.

### 4.2 Molecular markers

Recent molecular marker research on the chrysanthemum cold tolerance is mainly based on traditional molecular markers to help elucidate the related quantitative genetic mechanism (Zhang et al., 2010; Ma et al., 2018; Ao et al., 2019; Xu et al., 2019), but the potential for molecular marker-assisted breeding is limited. To date, many studies on molecular markers of chrysanthemum cold tolerance have mainly focused on the localization of LT50 and phenotypic traits related to cold tolerance. Ma (2017) used character-marker variance analysis, combined with the characteristics of high sequence-related amplified polymorphism (SRAP) marker polymorphism, to study LT50 molecular markers of the cold tolerance of the F_1_ chrysanthemum hybrid at different growth stages and obtained a total of 97 SRAP marker sites significantly related to the cold tolerance of chrysanthemum through one-way ANOVA. The contribution rates of a single marker site to coldtolerance variation ranged from 4.32% to 14.99%. In particular, the contribution rates of X-M18E7-133, X-M3E16-187, M-M13E19-135, XM-M21E15-143 and X-M3E16-187 were all above 10%, and the contribution rate of X-M3E16-187 was the highest (14.99%). These results demonstrate that cold tolerance chrysanthemum is a complex quantitative trait.

With the help of molecular marker technology, chrysanthemum varieties with strong cold tolerance can be screened using marker sites, and the phenotypic trait marker sites related to cold tolerance can also be unearthed, which will broaden the direction of research on cold tolerance in chrysanthemum. Yang et al. (2020) found three molecular markers (E11M24-3, E11M24-4 and E11M24-5) that were significantly correlated with the cold tolerance of tongue flowers by correlation analysis of the LT50 of 83 chrysanthemum tongue flowers, among which the E11M24-4 marker was also correlated with two phenotypic traits, the capitular diameter and the stem diameter. In addition, four cold-resistant chrysanthemum varieties (Qx034, Qx138, Qx145, and QD048) were identified. Xu et al. (2019) evaluated the LT50 values in the leaves of 83 cut chrysanthemum varieties at the seedling stage using electrical conductivity combined with a logistic equation, and 11 excellent allelic variation sites related to cold tolerance were detected through association analysis, among which 8 variation sites showed enhanced effects. The cold tolerance of cultivars with E7M12-13 was significantly higher than that of cultivars without E7M12-13, and six varieties with strong cold tolerance, namely Nannongjin lemon, Qx097, QD028, Qx049, Qx153 and Qx008, were found according to the synergistic sites. Xu et al. also found that three markers, E2M16-2, E2M16-1 and E11M23-14, related to cold tolerance were related to the flowering time, flower neck length and leaf edge serrated phenotypic traits, respectively, indicating that there was a certain correlation between cold tolerance and phenotypic traits in chrysanthemum.

In conclusion, the LT50 molecular marker is mainly used to study the tolerance in chrysanthemum, and chrysanthemum varieties with strong cold tolerance can be screened and identified using the marker sites. In addition, the phenotypic traits related to cold tolerance can be screened by molecular marker technology, laying a foundation for the breeding and application of cold-resistant chrysanthemums.

### 4.3 QTL mapping

With the development of molecular marker technology, QTL mapping, based on linkage mapping, provides an important means for genetic research on plant cold tolerance. In terms of cold tolerance, Ma (2017) further carried out QTL mapping of chrysanthemum cold tolerance based on the SRAP marker study and the genetic maps of ‘Nannong Xuefeng’ and ‘Mengbai’ and detected a total of 15 QTLs that were significantly correlated with chrysanthemum cold tolerance at four stages: the seedling stage, bud stage, full flower stage and foot bud stage. The contribution rate of a single QTL to explaining cold-tolerance variation ranged from 6.47% to 68.89%, and most of the QTLs were detected at more than two growth stages. In addition, the phenotype of a cold tolerance QTL obtained by this method was stable at different growth stages and was less affected by the environment, while other QTLs detected at a single growth stage were more affected by the environment, which further explained the correlation with cold tolerance at different growth stages. These findings also improved the possibility and predictability of selecting good genotypes for target traits in breeding. In conclusion, QTL mapping based on genetic maps is more convenient, intuitive and stable than traditional molecular markers, but QTL mapping requires genetic maps of hybrid parents. At present, there are few studies on the construction of genetic maps of hybrid parents of cold-resistant chrysanthemums, which can be further studied.

### 4.4 Genome-wide association studies

With the development of high-throughput sequencing technology, SNP markers based on genome-wide association studies (GWAS) have been applied to genetic studies of plant stress tolerance. At present, there are relatively few genome-wide association analyses of chrysanthemum cold tolerance. Fan et al. (2019) used 58 cut-flower chrysanthemums as materials for whole-genome association analysis to identify 24 SNP loci that were significantly correlated with cold tolerance at the foot bud stage, bud stage, leaves at the full flowering stage and tongue flowers at the full flowering stage and compared and analysed the specific locus amplified fragment (SLAF) tag sequences wherein significant SNP loci were located using the chrysanthemum transcriptome database. Five candidate genes were identified, among which CL2042.Contig4_All and Unigene40993_All may be associated with low-temperature stress. In conclusion, SNP markers based on GWAS are more convenient and accurate than traditional molecular markers and QTL mapping studies and effectively broaden the genetic research basis of chrysanthemum cold tolerance. However, there are too few relevant studies, and more in-depth research is needed.

There are relatively few forward genetics studies on the cold tolerance in chrysanthemum, mainly focusing on genetic analysis, molecular markers, QTL mapping and genome-wide association analysis. QTL mapping and genome-wide association analyses of chrysanthemum cold tolerance are less common than those of other plants. However, Ma (2017) analysed hybrid progeny through a mixed genetic model using the major gene plus multigene method and QTL mapping and found differences in the results of the two methods of analysis for major gene mapping at the bud stage, indicating that different methods should be used for comparative genetic analysis. In addition, research on cold tolerance using forward genetics methods are insufficient, and it is necessary to combine genomics, transcriptomics, proteomics and other omics approaches to further study the genetic mechanisms of chrysanthemum cold tolerance from the perspective of reverse genetics, which also lays a foundation for the discovery of chrysanthemum cold tolerance genes and transcription factors and their application in chrysanthemum cold tolerance breeding.

## 5 Research advance on the molecular mechanisms of cold tolerance in chrysanthemum

In recent years, the molecular mechanisms of plant responses to low-temperature stress have been extensively studied. The expression of cold response genes can be induced under low-temperature conditions, which can reduce the damage to plants caused by low-temperature stress and thus enhance the tolerance of plants to low-temperature stress. Many studies on the molecular mechanisms of cold tolerance in chrysanthemum mainly focus on functional genes and regulatory genes, which play an important role in gene expression and signal transduction.

### 5.1 Functional genes

Functional genes are genes directly related to the improvement of plant cold tolerance, such as cold-induced genes, fatty acid desaturase genes and antioxidant oxidase genes, which have protective effects on the cell membrane (Huang and Chen, 2011; Yao, 2013). Studies on functional genes of cold tolerance in chrysanthemum have mainly focused on photosynthetic system genes and fatty acid desaturation genes.

#### 5.1.1 Photosynthetic system genes

Light harvesting a/b-binding protein *CAB* gene in PSII of higher plants belongs to the gene of photosynthetic system (Raghvendra, 2001), and *CAB* can form light-trapping pigment protein complex with chlorophyll (Jansson, 1999). The light-trapping pigment complex can capture light energy and transfer it to the reaction center, maintain the thylakoid membrane structure, and regulate the energy distribution of PSI and PSII. Light-harvesting complex I chorophyll a-b binding protein (LHCI) is found on the thylakoid membrane (Green et al., 1991; Yu et al., 2001), including Lhca1, Lhca2, Lhca3 and Lhca4. In a study on the cold tolerance of the autumn chrysanthemum variety ‘Tangyu Jinqiu’, Zhao (2019) found that the *CmCAB* and *CmLhca2* showed a certain correlation with several chlorophyll fluorescence parameters. With the decrease in the stress temperature, the expression of the *CmCAB* gene was downregulated, and the number of pigment protein complexes was reduced, which weakened the absorption and transformation of light energy by PSII, reduced the degree of light inhibition, and alleviated the damage to PSII. The expression of the *CmLhca2* gene was continuously downregulated with decreasing stress temperature, and the LHCI content was reduced to alleviate the photoinhibition and damage to PSI caused by low temperature. However, abnormal changes occurred in the M_R_/M_R0_ curve at -4°C, indicating that the low temperature caused relatively serious damage to PSI.

In summary, studies on the photosynthetic system genes *CmCAB* and *CmLhca2* of chrysanthemum found that the expression of these two genes was downregulated with decreasing temperature under low-temperature stress, thus reducing the degree of low-temperature damage to PSII and PSI. Various photosynthetic parameters in the reaction process of PSII and PSI are closely related to the chrysanthemum cold tolerance. *CmCAB* and *CmLhca2* can indirectly affect chrysanthemum cold tolerance.

#### 5.1.2 Fatty acid desaturase gene

When plants are in a lower-temperature environment, the activity of desaturase increases, resulting in an increase in the content of unsaturated fatty acids, allowing the cell membrane to still maintain its original liquid state at a lower temperature. At low temperature, fatty acid desaturase has a certain protective effect on plant cells. According to the different substrates, vegetable fatty acid desaturases can be divided into acyl-ACP desaturases and acyl-ester desaturases.

##### 5.1.2.1 Δ9 Stearoyl-ACP desaturase gene

Acyl-ACP desaturase in plants is mainly located on plastosomes and is water soluble (Li et al., 2009), and it is the only known soluble desaturase system. Among these enzymes, Δ9 stearoyl-ACP desaturase (SAD) is the most extensively tested and most common acyl-ACP desaturase. Nie et al. (2020) studied the chrysanthemum ‘Jinlong Tengyun’ and found that low-temperature stress changed the expression levels of the *CmSAD*, *CmFAD2* and *CmFAD7* genes, which further promoted an increase in the unsaturated fatty acid mass fraction and played a certain protective role in PSII. Miao et al. (2022) found that the *CmSAD* gene of the chrysanthemum ‘Tangyu Jinqiu’ was significantly upregulated at low temperature; however, the stearic acid content did not significantly change in the cell membrane, suggesting that stearic acid synthesis was not a single pathway. In addition, in the thylakoid membrane, *CmSAD* gene expression was significantly negatively correlated with the C18:0 content and positively correlated with the C18:1 and C18:2 contents. This gene has a more direct regulatory effect on lipid unsaturation of the chrysanthemum thylakoid membrane; the expression of the *CmSAD* gene is upregulated, the concentration of the catalytic substrate is decreased, and the content of unsaturated fatty acids is increased.

##### 5.1.2.2 Acyl-lipid desaturase gene

Acyl-lipid desaturases can be divided into two categories corresponding to different substrates: ω-6 and ω-3 fatty acid desaturases. The main ω-6 fatty acid desaturases are mainly encoded by *FAD2*, *FAD4* and *FAD6*, and the main ω-3 fatty acid desaturases are encoded by *FAD3*, *FAD7* and *FAD8*. Many studies have mainly focused on *FAD2* and *FAD7*. Wang et al. (2014) cloned the *CmFAD7* gene using the chrysanthemum ‘Starlight Bright’ as the material and found that under different treatment temperatures, the expression levels of the *CmFAD7* gene in leaves were higher than those in roots. With decreasing temperature, the expression level of *CmFAD7* in leaves first increased and then decreased, while that in roots peaked peak at 5°C and then significantly decreased. Li et al. (2015) also used the chrysanthemum ‘Starlight Bright’ as the material to study differences in the expression levels of cloned *CmFAD7* under low-temperature stress in leaves and roots by a real-time fluorescent quantitative PCR method. They found that under low-temperature stress, the expression of *CmFAD7* in the leaves of the chrysanthemum was upregulated and was higher than that in roots, and the content of linolenic acid was increased. When the temperature was reduced to -8°C, the expression levels in leaves and roots were lower. Using the chrysanthemum ‘Tangyu Jinqiu’, Zhao et al. (2019) found that the expression levels of the *CmFAD2* and *CmFAD7* genes were generally upregulated under low-temperature stress, which effectively improved the unsaturation of thylakoid membrane lipids and enhanced the stability of the thylakoid membrane.

In summary, through the mining of desaturase genes of different chrysanthemum varieties, it was found that different genes had different effects on different parts of chrysanthemum plants, but the mode of action was the same, and they all directly regulated the membrane desaturase content to cope with low-temperature stress.

### 5.2 Regulatory genes

Regulatory genes mainly regulate the expression of cold tolerance genes, cold signal transduction and other processes to improve plant cold tolerance. Transcription factors (TFs) are protein molecules located in the nucleus that can specifically interact with cis-acting elements of gene promoter regions, and their main function is to activate or inhibit gene transcription (Jain et al., 2008). In recent years, a series of transcription factors families related to plant cold tolerance, including the MYB, bZIP and TCP families, have been isolated and identified, and the regulatory mechanisms of related cold-tolerance genes have been studied (Bhattacharjee, 2005).

#### 5.2.1 MYB transcription factors

MYB transcription factors contain highly conserved DNA-binding domains, typically consisting of 1 ~ 4 imperfectly replicated R junctions and 50 to 52 amino acid residues (Riechmann et al., 2000). MYB transcription factors play an important role in plant responses to cold stress, but the underlying mechanisms remain unclear. Yang et al. (2022) isolated the cold-induced R1-MYB transcription factor *DgMYB2* from *Chrysanthemum* and found that its overexpression enhanced cold tolerance, while antisense inhibition of *DgMYB2* resulted in decreased cold tolerance. An electrophoretic mobility shift assay (EMSA), chromatin immunoprecipitation (ChIP), luciferase complementary imaging (LCI) analysis and a dual-luciferase reporter gene assay (DLA) showed that *DgMYB2* directly targeted the MYB-binding site (CAACCA) in the *DgGPX1* promoter, increasing *GPX* enzyme activity and reducing ROS accumulation, thereby improving cold tolerance of daisy. He (2019) cloned *DgMYB1* from *Chrysanthemum* ‘Shenma’, transformed chrysanthemum by the *Agrobacterium*-mediated method to obtain two chrysanthemum lines, OE-18 and OE-25, with *DgMYB1* gene transfer and tested the cold tolerance of the two transgenic plants. The overexpression of *DgMYB1* in chrysanthemum decreased the REC and MDA content, increased the activities of SOD, POD and CAT, and increased the contents of SS, SP and Pro. These results indicate that *DgMYB1* can improve the tolerance of transgenic chrysanthemum to low-temperature stress, to a certain extent; it can be used as an excellent cold-tolerant plant breeding gene and can provide an effective gene reserve for new cold-tolerant chrysanthemum varieties.

#### 5.2.2 bZIP transcription factors

In higher plants, the bZIP transcription factor family has a positively charged, highly conserved domain consisting of 60-80 amino acids, with an acid-activated base region at its N-terminus (Cao et al., 2012). bZIP transcription factors play a very important role in abiotic stress, such as low-temperature stress, but there are relatively few studies on the role of bZIP transcription factors in chrysanthemum cold tolerance. Bai et al. (2022) found that overexpression of *DgbZIP3* resulted in increased cold tolerance in chrysanthemum, while antisense inhibition of *DgbZIP3* resulted in decreased cold tolerance. EMSAChIP, LCI assay DLA showed that *DgbZIP3* could directly bind to the *DgPOD* promoter and activate its expression. *DgbZIP2* was identified as a *DgbZIP3*-interacting protein by yeast two-hybrid, coimmunoprecipitation, LCI and bimolecular complementary fluorescence assays. Overexpression of *DgbZIP2* led to increased cold tolerance, while antisense inhibition of *DgbZIP2* led to decreased cold tolerance. ChIP-qPCR showed that *DgbZIP2* was highly enriched in the *DgPOD* promoter. The DLA, EMSA and LCI assays further indicated that *DgbZIP2* could not directly regulate the expression of *DgPOD*. Wang (2019) cloned *DgbZIP2* from the chrysanthemum ‘Shenma’ and obtained transgenic plants. The study found that the expression of *DgbZIP2* was induced and upregulated under low-temperature stress, and the expression level was higher in leaves. In addition, the phenotype and physiological index of cold tolerance of the transgenic plants were examined. The lodging, wilting and survival rates of the chrysanthemums overexpressing *DgbZIP2* were higher than those of wild-type chrysanthemums at low temperature; the activities of SOD, POD and CAT were increased, the content of MDA was decreased, and the SS, SP and Pro contents were increased. These results indicated that *DgbZIP*2 is a positive regulator of low-temperature stress, and the transgenic chrysanthemum plants had strong cold tolerance.

#### 5.2.3 TCP transcription factors

During plant growth and development, long noncoding RNAs (lncRNAs) are at the core of gene regulatory networks, which are related to plant development, nutrient metabolism, and biological and abiotic stress processes. Although there are few studies on their functional mechanisms, many lncRNAs can act as scaffolds to mediate chromatin remodelling and histone modification to affect downstream gene expression (Lucia and Dean, 2011; Heo and Sung, 2011; Kim and Sung, 2017). Li et al. (2022) discovered a lncRNA, named *DglncTCP1*, that regulated transcribed *DgTCP1* from the TCP transcription factor. In response to low-temperature stress, the overexpression of *DgTCP1* and *DglncTCP1* enhanced chrysanthemum cold tolerance. In addition, LCI and DLA experiments showed that overexpression of *DglncTCP1* upregulated the expression of *DgTCP1* and that *DglncTCP1* may play a cis-regulatory role in the cold-tolerance regulation of *DgTCP1*. Using ChIP-qPCR, *DglncTCP1* was found to be a scaffold, and the histone-modified protein *DgATX* was recruited to *DgTCP1* to increase the level of H3K4me3, thus activating *DgTCP1* expression. *DgTCP1* can directly target *DgPOD*, promote its expression and reduce ROS accumulation, thus improving chrysanthemum cold tolerance.

Furthermore Huang et al. (2020) found that the acylation degree of *DgTIL1* increased at low temperature, and the interaction degree with *DgnsLTP* was enhanced, which was conducive to the increase of POD activity in chrysanthemum and the ability of chrysanthemum to resist low temperature. Bai et al. (2021) found that the expression of resistance related genes such as *DgCOR413*, *DgDREBa*, *DgCSD1* and *DgCSD2* in plants overexpressed with zinc finger protein *DgC3H1* increased, and the resistance to low temperature was significantly higher than that of wild type chrysanthemum.

In summary, through the molecular mechanisms by which the MYB, bZIP and TCP transcription factors regulate chrysanthemum cold tolerance, it was found that the transcription factors could activate the expression of target genes and increase catalase activity to reduce the accumulation of ROS, thereby indirectly improving chrysanthemum cold tolerance. Research on these three transcription factors continues. In particular, *DgMYB2* was only studied for its regulation of the downstream target gene *DgGPX1*. *DgbZIP3* was studied not only for its regulation of the downstream target gene *DgPOD* but also for the interaction between *DgbZIP3* and *DgbZIP2*. *DgbZIP2* could not directly activate the expression of *DgPOD* but coregulated *DgPOD* with *DgbZIP3*. However, the *DgTCP1* study introduced a new idea. Starting from the regulation of target genes by the lncRNA, it was found that *DglncTCP1* could act as a scaffold to mediate the recruitment of *DgATX* to *DgTCP1*, thereby increasing the level of H3K4me3 on *DgTCP1* and activating its expression. In addition, *DgTCP1*, as a transcription factor, can bind to the promoter of the target gene *DgPOD* to regulate its transcription, which provides a reference for the study of multiple transcription factors in one direction. Furthermore, *DgMYB1* and *DgbZIP2* were used in transgenic chrysanthemum experiments and were verified to have positive regulatory effects on low-temperature stress at the phenotypic and physiological levels.

There are relatively few forward genetics studies on on cold tolerance in chrysanthemum, mainly focusing on genetic analysis, molecular markers, QTL mapping and genome-wide association analysis. QTL mapping and genome-wide association analysis of chrysanthemum cold tolerance are less than those of other plants. However, Ma Jie analysed a hybrid progeny through the mixed genetic model using the major gene plus multigene method and QTL mapping and found differences in the results of the two methods of analysis for major gene mapping at the bud stage, indicating that different methods should be used for comparative genetic analysis. In addition, research on cold tolerance and forward genetics methods are insufficient, and it is necessary to combine genomics, transcriptomics, proteomics and other omics approaches to further study the genetic mechanisms of chrysanthemum cold tolerance from the perspective of reverse genetics, which also lays a foundation for the discovery of chrysanthemum cold tolerance genes and transcription factors and their application in chrysanthemum cold tolerance breeding.

There are relatively few studies on the molecular mechanism of cold tolerance in chrysanthemum, and they have mainly focused on functional genes and regulatory genes. Research on photosynthetic system genes in functional genes is still relatively limited, and further research is needed. Studies on desaturase genes are relatively sufficient, but the types and quantities of desaturase need to be further explored. In terms of regulatory genes, the functions of related transcription genes are mainly analysed from transcription factors, and related genes can also be cloned from chrysanthemum varieties to identify cold tolerance through transgenic plants. However, the number of transcription factors related to cold tolerance is still relatively small and needs to be further explored. These research findings lay a foundation for the breeding and application of new chrysanthemum varieties with cold tolerance and provide new breeding ideas.

## 6 Research progress on the breeding methods of cold tolerance in chrysanthemum

With the research and analysis of phenotypic evaluation indices, physiological mechanisms, forward genetics and molecular mechanisms, an increasing number of breeding methods are emerging for chrysanthemum. The demand for cold-resistant herbs in all four seasons is also increasing in urban landscaping, and the breeding of new cold-resistant chrysanthemum varieties has become an area of intense interest. Many studies on breeding methods for cold tolerance in chrysanthemum have mainly focused on crossbreeding, radiation mutagenesis breeding, selective breeding and genetic engineering breeding.

### 6.1 Crossbreeding

Crossbreeding is a conventional method of flower breeding and one of the most widely used and effective breeding methods in China and around the world. The use of heterosis is one of the most important methods in the hybrid breeding of chrysanthemum, and the probability of hybridization in the hybrid progeny population is greater with a greater genetic locus difference between parents (Zhou and Duan, 1995; Chen et al., 2009). Studies on crossbreeding of cold tolerance in chrysanthemum have mainly focused on the selection of distant crosses, intergeneric crosses and heterosis.

For distant crossbreeding, Zhu et al. (2012) crossed ‘Zhongshan Jingui’ as the maternal parent and *Ajania nematoloba* as the male parent, and the cold tolerance of the F1 hybrid was significantly improved, but the ornamental value was worse than that of ‘Zhongshan Jingui’, and further improvement was needed. Afterwards, Zhu et al. used the F1 hybrid as the parent and ‘Zhongshan Jingui’ as the recurrent parent and obtained 17 backcross lines. The backcross offspring had stronger cold tolerance than ‘Zhongshan Jingui’ and not only inherited the cold tolerance characteristics of *Ajania nematoloba* but also improved the ornamental quality of the distant hybrid offspring. Deng et al. (2011) carried out intergeneric hybridization using embryo culture technology with ‘Zhongshan Jingui’ as the maternal parent and a wild chrysanthemum as the paternal parent and obtained five intergeneric hybrids. The determination of the LT50 and Pro and MDA contents showed that the hybrid offspring had the same cold tolerance characteristics as their parents, with high cold tolerance. In terms of heterosis, Ma (2017) used ‘Nannong Xuefeng’, which has strong cold tolerance, as the maternal parent and ‘Mengbai’, which has poor cold tolerance, as the paternal parent of the F_1_ chrysanthemum hybrid in full bloom and found that the cold tolerance of the chrysanthemum tongue flowers had certain maternal inheritance. In addition, the cold tolerance of the tongue flowers was related to the number of days in full bloom. Therefore, varieties with strong cold tolerance and late flowering should be chosen as the mother. This finding provides a reference for the cultivation of strong cold tolerance lines.

In conclusion, crossbreeding chrysanthemums for cold tolerance has achieved certain research results in distant crosses, intergeneric crosses and heterosis, but it is still necessary to further use a variety of chrysanthemum materials to cultivate more chrysanthemum varieties with strong cold tolerance.

### 6.2 Mutagenesis breeding

Chrysanthemums are genetically highly heterozygous, and evolution proceeds from low to high allopolyploidy, which easily leads to complex changes in genetic factors and is more suitable for radiation mutagenesis. Radiation breeding has the characteristics of high mutagenesis efficiency and good repeatability (Techarang et al., 2018; Khitka et al., 2021) and has become an important means of chrysanthemum genetic improvement. To date, research on radiation breeding for cold-resistant chrysanthemum has mainly focused on different mutagenesis methods. Fu and Zheng (1994) treated materials selected for mutagenesis with ^60^Co and selected eight new varieties of ‘Hanju’, whose natural flowering period was from late November to early January of the following year and which could flower normally at -2°C to -5°C with rich flowers of relatively high ornamental value. Ueno et al. (2003) used carbon ion-beam radiation produced by a TIARA accelerator to mutate leaves. After culturing and screening, 66 identifiable mutant materials, including those with early flowers, late flowers, a reduced number of axillary buds, and flowering at low temperature, were isolated from 13077 M1 plants. Ueno et al. (2013) also used this radiation source to radiate ‘Shenma’ plants and obtained new chrysanthemum varieties ‘Xinshen’ and ‘Xinshen 2’, which had a reduced number of axillary buds and could bloom normally under low-temperature conditions. In conclusion, there are still few studies on radiation breeding of cold-resistant chrysanthemums, which is possibly related to the uncontrollability of radiation mutagenesis, and further practical studies are needed.

### 6.3 Selective breeding

Selective breeding is the process of artificially selecting and propagating the characteristics of natural variation or artificial pollination variation in the planting process to cultivate new strains, which is one of the most important means of conventional plant breeding. Many studies in chrysanthemum have mainly focused on character selection, physiological indices and molecular marker-assisted breeding. In terms of character selection, Fu (1998) selected new cold chrysanthemum varieties with strong cold tolerance, ‘Wanyu’, ‘Wanfenhe’, ‘Hongwanying’ and ‘Huangwanying’, based on the morphological characteristics of the pollen. Miao et al. (2013) obtained a new chrysanthemum variety ‘Yanzhilu’ with strong cold tolerance after seed selection from the budding materials of the chrysanthemum variety ‘Italian Red’. However, this method required certain experience, and there were certain accidental phenomena, which often resulted in the selected plants not carrying the target traits.

Regarding the selection and breeding of physiological indicators, a previous review of physiological indicators reported that SOD and POD activities and the MDA, SS, and Pro contents and other chrysanthemum physiological indicators can be determined (Xu et al., 2010; Li, 2010; Li et al., 2009; Cheng et al., 2018), and plants with strong cold tolerance can be evaluated after analysis. Compared with conventional selection and breeding methods based on traits, selection results based on physiological indicators are more accurate, but certain experimental conditions are needed. Marker-assisted selection (MAS) is a breeding technique that uses closely linked or coisolated markers to select individuals for target traits (genes), regardless of gene expression, growth stage and environmental factors (Wang L. S. et al., 2006), which can greatly shorten the breeding time. MAS has become a research focus in the field of plant genetics and breeding (Klie et al., 2016; Cobb et al., 2019). For MAS of chrysanthemum cold tolerance, Yang et al. (2017) obtained three molecular markers through correlation analysis of chrysanthemum cold tolerance and LT50 and then selected four chrysanthemums with strong cold tolerance. Ma conducted a QTL mapping study on the cold tolerance data for the ‘NannongXuefeng’ × ‘Mengbai’ F_1_ generation and found that the contribution rates of qBdsCTM33 and qFfsCTM33 on the M33 linkage group to cold tolerance at the bud and flowering stages were 65.78% and 68.89%, respectively. These two QTLs may be the main genes controlling cold tolerance, which can be verified in multiple environments in future studies and applied to the selective breeding of cold-tolerant chrysanthemums. Compared with conventional character selection breeding and physiological selection breeding, molecular marker-assisted breeding can select the target character more accurately. In conclusion, with the in-depth study of phenotypes and physiological and molecular mechanisms related to chrysanthemum cold tolerance, an increasing number of breeding methods have appeared, and they are faster and more accurate.

### 6.4 Genetic engineering breeding

Genetic engineering is based on the theory of molecular genetics and adopts various biotechnological methods to introduce and integrate foreign or original genes with different functions into plant cells and achieve effective expression of functional genes through transgenic means for the purpose of targeted improvement of plant quality and creation of new varieties (Zhang, 2013). Many studies in chrysanthemum have mainly focused on agrobacterium-mediated, asexual reproduction and gene recombination. Wu (2006) introduced the PEAMT gene into chrysanthemum using *Agrobacterium*, which increased the content of betaine in the plant, thus greatly enhancing its salt and cold tolerance. Hong et al. (2006) introduced the stress-induced transcription factor *DREB1A* into the ‘Fall Color’ groundcover chrysanthemum through the *Agrobacterium tumefaciens*-mediated method and somatic embryoblast pathway using the *AtDREB1A* transformation vector driven by 35S and rd29A as promoters, respectively, to make the transgenic plants more cold resistant. Chen et al. (2012) verified the tolerance of chrysanthemum plants with a transferred *CdICE1* gene to abiotic stresses, such as low temperature, drought and salt stress, and the results showed that the tolerance of the transgenic chrysanthemum plants to abiotic stresses was significantly improved. In terms of asexual reproduction, Li C. et al. (2010) observed an asexual progeny from the transfer of the *AtDREB1A* gene to ‘Fall Color’, studied by Hong et al. (2006), and found that the progeny plants obtained through tissue culture expansion had a certain cold tolerance, and their ability to overwinter in the open field was also significantly improved. In terms of gene recombination, Li et al. (2022) knocked out the *DgTCP1* gene in chrysanthemum using the CRISPR/Cas9 system and found that the cold tolerance of the *DgTCP1* mutant plants decreased, while that of chrysanthemum transgenic plants overexpressing *DgTCP1* increased, providing an important reference for improved low-temperature tolerance breeding. Yang (2021) transferred the *DgnsLTP* overexpression vector into wild type chrysanthemums by Agrobacterium mediated method, and obtained the *DgnsLTP* overexpression line *35S: DgnsLTP-1*, *35s: DgnsLTP-3*, and found that they had higher survival rate and stronger resistance to low temperature stress than wild type chrysanthemums. In summary, genetic engineering breeding of cold tolerance chrysanthemum is controllable, and certain research results have been achieved in the understanding of the physiological, genetic and molecular mechanisms in chrysanthemum. However, overall, genetic breeding research on cold tolerance chrysanthemum is still relatively limited, and further research is needed to meet the market demand for cold-resistant chrysanthemums.

There are relatively few studies on breeding methods for cold tolerance in chrysanthemum, mainly focusing on crossbreeding, mutagenesis breeding, selection breeding and genetic engineering breeding. Compared with the other three breeding methods, the research results for mutagenesis breeding are still insufficient, which is possibly related to the high genetic heterozygosity of chrysanthemum itself and the uncontrollability of mutagenesis breeding. More radiation materials and radiation technology should be used for mutagenesis breeding research. In addition, with the rapid development of various omics technologies, such as genomics, epigenomics, transcriptomics, proteomics, metabolomics and phenomics, and the significant reduction in breeding costs, multidimensional and multiomics research on chrysanthemum breeding can create better cold-resistant chrysanthemum varieties to meet the needs of scientific research and landscaping.

## 7 Conclusion and future prospects

In terms of chrysanthemum phenotype, characteristics such as foot buds, florescence, REC, LT50, and anatomical structure are important indicators for evaluating chrysanthemum cold tolerance. The quantity of foot buds, rooting rate, and root length can be visually observed in the field to assess the strength of chrysanthemum cold tolerance. REC generally increases gradually with decreasing temperature. Combining REC with the Logistic equation calculates the LT50, which directly reflects cold tolerance strength. Leaf anatomical structure can reflect chrysanthemum response to temperature changes, but observing the structure requires precise instruments and some anatomical experience, making it relatively challenging. Furthermore, there are discrepancies in the research results of cold tolerance and florescence in chrysanthemum, as shown by Li et al. (2013), Li (2010), and Xu and Chen (2008). The phenomenon of early-flowering varieties having stronger cold tolerance and late-flowering varieties having weaker cold tolerance needs further analysis and validation.

In terms of physiological mechanisms of chrysanthemum, there are various physiological indicators for cold tolerance identification, including the biological membrane system, protective enzyme system, osmotic regulatory substances, photosynthesis, and hormones. However, chrysanthemum research mostly focuses on the relationship between individual physiological indicators and cold tolerance. Therefore, it’s crucial to select effective physiological indicators for rapid and accurate cold tolerance identification. Many scholars have started using multiple indicators to comprehensively evaluate cold tolerance (Xu et al., 2009). Methods like membership functions and correlation analysis have been employed by Xu et al. (2010), Bai et al. (2013), and Qiao et al. (2023) to comprehensively assess the SOD, POD, and CAT activities activity and the MDA, SS, SP and Pro contents of different groundcover chrysanthemums, and ranked the strength of cold tolerance of different varieties. Therefore, establishing a reliable model for chrysanthemum cold tolerance evaluation through multiple physiological indicators is of significant importance for breeding new cold tolerance chrysanthemum varieties and evaluating cold tolerance across multiple varieties.

In the field of forward genetics, advancements have been made from traditional genetic analysis to molecular markers, QTL mapping, and GWAS. This includes processes like segregation ratio analysis, mixed inheritance model analysis, molecular markers, constructing hybrid parental genetic maps, and multi-variety association analysis. These processes progressively deepen and enhance the accuracy and efficiency of gene determination for traits. Regarding molecular mechanisms, chrysanthemum cold tolerance is controlled by polygenic effects. Cold tolerance genes mainly include functional genes directly related to cold tolerance and regulatory genes controlling cold tolerance gene expression and signaling. Functional genes include photosynthesis system genes and fatty acid desaturase genes, although they are less studied in chrysanthemum cold tolerance research. Regulatory genes, particularly transcription factors, are gaining attention. Many transcription factors associated with cold tolerance, such as AP2/EREBP, WRKY, MYB, bZIP, TCP, NAC, and Zinc-finger (Riechmann et al., 2000; Zhu et al., 2010; Iwase et al., 2011), have been identified. However, only MYB, bZIP, and TCP transcription factors have been identified in chrysanthemums so far, indicating the need for further research to discover other transcription factors.

In terms of chrysanthemum breeding, hybrid breeding remains the most commonly used method, involving interspecific hybridization, distant hybridization, and transgenic and non-transgenic hybridization to produce cold tolerance varieties. Mutational breeding is also an important method for genetic improvement of chrysanthemum, offering advantages such as rapid results. However, it comes with uncertainties and potential issues with inheritance due to chrysanthemum high genetic heterogeneity, as well as the mutagenic materials and techniques used. Selection breeding has evolved from traditional trait selection to molecular marker-assisted selection, enabling precise and rapid selection of target traits. Genetic engineering is a popular breeding approach, but its application in chrysanthemum cold tolerance research is relatively limited. This method involves introducing target genes into chrysanthemums through various techniques for validation, necessitating further in-depth research.

Based on the summarization and integration of research on chrysanthemum cold tolerance phenotype, physiological mechanisms, positive genetics, molecular mechanisms, and breeding methods, there remain numerous unresolved issues in the current study of chrysanthemum cold tolerance. The specific relationship between chrysanthemum cold tolerance and flowering stage requires further in-depth investigation. While LT50 may represent the results of most cold tolerance physiological indicators, within the intricate mechanism of low-temperature response, lipid metabolism also plays a crucial role. The relationship between fatty acid metabolism and LT50 needs further exploration. Epigenetics refers to the phenomenon where gene expression changes without altering the DNA sequence and is closely related to changes in external environmental conditions, making it suitable for studying chrysanthemum cold tolerance (Wang et al., 2022). Research on antifreeze protein genes (AFPs) has made significant progress in other plants but remains unexplored in chrysanthemum (Griffith et al., 1992; Urrutia et al., 1992; Duman and Olsen, 1993; Worrall et al., 1998; Yin et al., 2001). China possesses abundant chrysanthemum germplasm resources, and genetic breeding research has yielded fruitful results. However, research on breeding for cold tolerance is still insufficient. With the rapid development of high-throughput sequencing, proteomics, and metabolomics, the future holds the potential to better unveil chrysanthemum responses to varying levels of low-temperature stress at the RNA, protein, and metabolite levels. This includes discovering new genes, proteins, and differentially expressed metabolites related to cold tolerance, conducting multi-omics joint analyses, and systematically investigating the mechanisms underlying chrysanthemum cold tolerance. For example, chrysanthemum is rich in flavonoids, and the accumulation of flavonoids is positively correlated with the plant stress tolerance (Watanabe and Ayugase, 2015; Schulz et al., 2016; Zhou et al., 2021). While previous cold tolerance research in chrysanthemums mainly focused on single genes, cold tolerance is a quantitative trait controlled by multiple genes. In the future, employing the simultaneous transformation of multiple genes could enhance chrysanthemum cold tolerance.

## Author contributions

QC: Writing – original draft. KG: Writing – review & editing. YX: Writing – review & editing. YS: Writing – review & editing. BP: Writing – review & editing. DC: Writing – review & editing. CL: Writing – review & editing. XC: Writing – review & editing. HL: Writing – review & editing. CH: Writing – review & editing.
